# Effects of AR on Cognitive Processes: An Experimental Study on Object Manipulation, Eye-Tracking, and Behavior Observation in Design Education

**DOI:** 10.3390/s25061882

**Published:** 2025-03-18

**Authors:** Ju Yeon Kim, Jin Kyung Choi

**Affiliations:** Department of Interior Architectural Design, Soongsil University, 360 Sando-ro, Dongjak-gu, Seoul 06978, Republic of Korea; durslw@ssu.ac.kr

**Keywords:** spatial thinking, augmented reality, spatial visualization, design education, behavioral observation, eye-tracking

## Abstract

We investigate the impact of augmented reality (AR) on spatial reasoning and visualization skills in design education by analyzing users’ interaction behaviors with AR environments. Specifically, we examine how prior experience in spatial design influences engagement with AR tools. To this end, we compare the eye movement and operational behaviors of two groups: participants with a background in 3D space design (experimental group) and those without such experience (control group). Participants manipulated virtual objects within real-world environments at a 1:1 scale, and we analyzed interaction frequency, duration, and efficiency using frequency analysis and independent samples *t*-tests in SPSS 25. The results indicate that, although both groups engaged similarly with AR content, the experimental group demonstrated more precise manipulation and a deeper understanding of spatial relationships, leading to more efficient task completion. These findings suggest that AR-based learning experiences should be tailored to learners’ spatial reasoning abilities to maximize educational benefits. Consequently, educators should design adaptive AR environments that accommodate diverse skill levels to ensure optimized learning outcomes in spatial design education.

## 1. Introduction

Emerging technologies have become pivotal in enhancing human sensory and cognitive capabilities and have been introducing innovative methodologies and tools across various domains. Among these advancements, augmented reality (AR) has emerged as a transformative visualization technology with multifaceted applications. AR enables users to experience an overlay of virtual environments on the real world, thereby seamlessly integrating virtual objects into the physical environment in real time [1]. This integration of real and virtual environments offers unparalleled realism, immersive experiences, and intuitive interaction mechanisms, increasingly supported by advancements in smart devices such as smartphones and tablets [2,3].

In the field of education, particularly in design learning, the adoption of AR has led to significant pedagogical shifts. Traditional design education, which has long relied on static, two-dimensional representations and conventional modeling techniques, is being transformed by AR’s ability to facilitate dynamic, interactive, and spatially immersive learning experiences. Recent studies have focused on understanding how AR-based learning environments enhance spatial reasoning, improve conceptual visualization, and foster user engagement by bridging the gap between theoretical knowledge and practical application.

- AR in Education: Enhancing Immersive Learning

In the education sector, AR is celebrated for its ability to provide immersive, interactive learning experiences that overcome traditional constraints and enhance conceptual understanding. Specifically, AR allows users to visualize complex concepts, conduct controlled field-based explorations, and engage with abstract phenomena that are otherwise difficult to replicate in conventional settings [4,5,6,7,8]. Furthermore, recent studies have highlighted the role of immersive AR in improving learning outcomes and engagement levels by fostering personalized, adaptive learning environments [9]. Beyond enhancing engagement, AR also contributes to the social sustainability of educational practices by supporting inclusive and accessible learning environments [10]. By integrating interactive digital elements, AR not only aids in the comprehension of scientific, mathematical, and artistic principles [11,12,13,14], but also promotes collaborative learning and knowledge-sharing through virtual interactions.

- AR in Architectural Design Education

AR has demonstrated transformative potential in architectural education by supporting incremental learning processes that enhance students’ spatial visualization and design cognition. This technology facilitates the comprehension and manipulation of three-dimensional (3D) objects, thereby offering students opportunities to develop critical skills in spatial reasoning and in the practical application of design principles [15,16]. This interactive learning process enables students to bridge the gap between theoretical knowledge and practical application by engaging in real-time experimentation and visualization in a controlled environment [7,17,18,19]. The integration of AR with advanced digital design tools, such as computer-aided design, Rhino, SketchUp, and Lumion, further enhances the design learning process. These tools enable students to conceptualize and manipulate 3D spaces more efficiently, reducing cognitive load and increasing design fluency [20,21]. In addition, AR applications in architectural education can extend beyond the classroom, allowing students to interact with urban-scale digital models and assess the perception of public spaces [22]. Such developments highlight the growing role of AR in not only refining design pedagogy but also influencing public space planning and user engagement in urban environments. These developments not only streamline the design process but also foster creativity and innovation among learners.

- Challenges in AR-Based Design Education

Despite the evident benefits of AR, challenges remain in optimizing its potential in design education. Cognitive overload is a significant issue, as learners must process and interpret complex 3D design outcomes while interacting with AR environments, and hence, a structured pedagogical approach is required to maximize learning efficiency and minimize cognitive fatigue. To address these challenges, it is necessary to develop innovative methods that leverage the immersive capabilities of AR to simplify and enhance spatial reasoning processes [23,24].

In this study, we investigate the impact of real-scale virtual objects in AR environments on spatial reasoning and visualization education. By focusing on user behavior and cognitive responses, we aim to uncover the mechanisms underlying the processing of visual information and decision-making in AR contexts. Specifically, we examine furniture arrangement in a virtual space as a case study to analyze user interaction and manipulation behaviors. By using eye-tracking technology and behavioral observation, this study provides insights into improving design-related problem-solving, enhancing user motivation, and fostering a deeper understanding of spatial visualization in AR-based educational settings. Its findings have the potential to contribute significantly to the refinement of AR applications in educational contexts, particularly in design and architectural training. By addressing current limitations and proposing innovative strategies, this study underscores the transformative power of AR in shaping the future of immersive and interactive learning environments.

## 2. Literature Review

### 2.1. Spatial Thinking and User-Centered Observational Research

Spatial thinking is a fundamental cognitive process that involves perceiving, analyzing, and interpreting spatial relationships to structure knowledge and solve problems. Han [25] described spatial thinking as a universal ability shaped by human development, emphasizing its role in organizing thoughts through spatial concepts, such as position, distance, and direction. Choi [26] further explained that spatial thinking is not innate but, rather, cultivated through consistent practice and exposure to real-world spatial environments, highlighting the role of educational and technological interventions in its enhancement. Newcombe [27] emphasized the application of spatial thinking in practical domains, asserting that individuals with advanced spatial reasoning can use tools and technologies to represent spatial concepts and make informed judgments about spatial data. Spatial visualization, a subset of spatial thinking, is particularly important in fields such as architecture, engineering, and interior design, in which professionals must interpret, manipulate, and predict spatial relationships between objects. Hsing et al. [28] and Zhou et al. [29] showed that spatial visualization skills can be systematically developed through targeted training programs that focus on mental imagery and object manipulation.

Technological evolution has significantly affected spatial thinking by introducing advanced tools, such as AR. This tool enables users to merge real-world environments with virtual overlays in real time and thus addresses the inherent limitations of traditional two-dimensional spatial representations [30,31]. This integration enhances users’ ability to visualize complex spatial relationships and fosters a deeper comprehension of spatial concepts. For instance, AR applications in architectural and design education have shown significant potential in facilitating incremental learning processes by allowing users to interact with and manipulate 3D models in immersive environments [32].

The National Research Council [33] identified three core components of spatial thinking: spatial concepts, spatial representation, and spatial reasoning. Han [25] elaborated on this framework, explaining that these components collectively enable the interpretation and application of spatial information.

- Spatial Concepts: These form the foundational understanding of spatial properties, such as dimensions, directions, and spatial relationships [34].
- Spatial Representation: This involves visualizing and sensorially manifesting spatial relationships through diagrams, models, and interactive tools.
- Spatial Reasoning: This cognitive process entails interpreting, predicting, and manipulating structured spatial data to solve problems and make informed decisions.

The integration of spatial thinking with user-centered observational research offers opportunities to investigate individuals’ perceptions and interactions with spatial elements in various contexts. Observational studies have provided insights into users’ behavior, preferences, and cognitive responses when they engage with spatial information and tools. Techniques such as eye-tracking and behavioral analysis have been employed to study the effectiveness of spatial visualization tools, including AR applications, in enhancing spatial reasoning and problem-solving capabilities [19,23]. Spatial thinking is a critical skill that underpins human interaction with spatial environments, and it enables individuals to understand and manipulate spatial relationships effectively. By fostering spatial visualization and integrating advanced technologies, such as AR, educators and researchers can enhance spatial reasoning skills and expand the potential of user-centered design approaches.

### 2.2. Educational Applications of Augmented Reality Technology

AR, an interactive technology that seamlessly overlays virtual objects onto the physical environment, offers heightened realism, immersion, and user engagement [35]. With its ability to integrate virtual and real-world elements in real time, AR has attracted significant attention, particularly in the field of education, for which it holds transformative potential. Shelton and Hedley [13] demonstrated that AR enables learners to acquire spatial information from both real and virtual environments simultaneously, thereby enhancing their cognitive ability to process and synthesize information. However, the extent to which different AR methods influence user cognition remains a critical research question. Although AR improves learning motivation, comprehension, and efficiency, challenges such as cognitive overload and technical limitations must also be addressed to fully leverage its benefits in educational contexts [36]. In educational settings, AR enhances students’ learning experiences by facilitating the visualization of abstract and complex concepts. By allowing them to interact with 3D representations, AR helps bridge the gap between theoretical knowledge and practical application, fostering active learning and deeper comprehension [37].

Among the various AR technologies utilized in education, marker-based and marker-less AR systems are two widely adopted approaches, alongside other methods such as projection-based and superimposition-based AR. Marker-based AR relies on physical markers, such as QR codes or printed images, to anchor virtual objects within real-world environments, whereas marker-less AR employs spatial recognition techniques to enable object placement without predefined markers [38]. Each approach presents distinct advantages and challenges in design education. Marker-based AR provides precise alignment of virtual objects with the physical space, enhancing spatial accuracy and stability. However, its dependency on external markers may limit flexibility in user interactions. In contrast, marker-less AR allows greater freedom in manipulating virtual objects, enabling learners to explore and experiment more dynamically. This flexibility, however, often comes at the cost of increased computational complexity and higher cognitive load, as users must mentally anchor and adjust objects without visual reference points.

Recent studies have supported this claim. For instance, Uriarte-Portillo et al. [9] found that learners with higher immersive profiles (those who engage more deeply with AR environments and exhibit greater presence and interaction) achieve better learning outcomes in AR-based educational settings, suggesting that personalized and adaptive AR experiences may further enhance learning efficacy. Similarly, in immersive learning contexts, AR empowers learners to solve real-world problems by simulating practical scenarios in controlled, yet realistic, environments. The literature has consistently highlighted the positive effects of AR on education. According to Park et al. [39], AR-based learning increases learners’ attention, engagement, and overall understanding of complex concepts, particularly in STEM (science, technology, engineering, and mathematics) education. AR provides interactive visualizations that improve problem-solving abilities and enable learners to explore topics from multiple perspectives.

In design education, the choice between marker-based and marker-less AR significantly impacts user interaction, cognitive load, and learning outcomes. Marker-based systems enhance precision and reduce cognitive demand by offering fixed reference points, making them suitable for structured educational tasks such as architectural modeling and engineering simulations [28]. Marker-less AR, on the other hand, fosters creative exploration by allowing students to experiment with spatial arrangements more freely. While this encourages deeper engagement with spatial reasoning, it may also require greater cognitive effort, particularly for novice users. As a result, the effectiveness of each AR system depends on the learning context and the proficiency level of the target audience. Martín-Gutiérrez et al. [38] emphasized the role of AR in promoting collaborative learning, wherein learners work together in shared AR environments, encouraging active participation and improving peer-to-peer interaction.

AR has significant implications not only for STEM education but also for spatial design and architecture education. As Đurić et al. [22] demonstrated, AR influences users’ spatial perception and interaction with digital environments, reinforcing its potential for training future architects and designers. Furthermore, AR contributes to bridging the gap between theoretical and practical education by enabling real-time experimentation and visualization. In design and architecture education, AR tools allow students to create, modify, and evaluate their projects dynamically, thereby fostering creativity and innovation [15]. The interactive and iterative nature of AR enhances learners’ spatial reasoning skills by allowing them to manipulate 3D objects and better comprehend spatial relationships.

Despite the advantages of AR, several challenges emerge when integrating it into education. The cognitive load associated with processing augmented and real-world information can sometimes overwhelm learners, particularly in complex educational contexts. In addition, financial constraints and the lack of skilled professionals to develop AR content remain significant barriers to its widespread adoption [36]. These limitations underscore the need for systematic instructional design and training programs to maximize the pedagogical value of AR.

Unlike traditional marker-based AR, which requires predefined physical markers to anchor virtual objects, this study utilizes a marker-less AR system that enables learners to interact freely with 3D-modeled virtual objects within real-world environments. By leveraging AR’s ability to dynamically integrate digital elements into physical spaces, this approach enhances user engagement and fosters a more intuitive spatial learning experience. Previous studies have demonstrated AR’s potential in design education by enabling learners to visualize and manipulate 3D objects within computer-generated. However, this study expands on that foundation by incorporating real-scale object interactions, allowing learners to engage with spatial environments in a more immersive and practical manner. The design of this system serves as a foundational proposal for bridging the gap between conventional 3D modeling software and real-world applications, maximizing AR’s extended benefits by reducing cognitive load while enhancing spatial cognition.

The integration of AR into curricula requires a strategic approach to maximize its benefits. In this regard, Alkhwaldi [10] emphasized the importance of designing immersive educational environments that align with social sustainability principles, ensuring that AR tools are accessible and beneficial to all learners. In particular, 3D AR content has proven to enhance learners’ comprehension of spatial and conceptual information that traditional two-dimensional representations fail to convey effectively [40,41]. By facilitating real-time decision-making, planning, and modification, AR enriches the learning process and aligns closely with modern pedagogical practices. As AR technology continues to evolve, it is crucial to establish frameworks for its effective implementation in educational contexts. This includes identifying the most suitable AR approach for different educational needs, balancing cognitive demands with learning benefits, and evaluating long-term impacts on student engagement and performance. When these frameworks and initiatives are addressed, AR can fulfill its potential as a transformative educational tool in the future of education.

### 2.3. Cognitive Characteristics Explored Through Eye-Tracking

Cognitive processes are foundational to human perception, attention, and memory and enable individuals to interact with their environment, make decisions, and achieve goals [42]. Although these processes are broadly understood in terms of their outcomes, significant challenges arise in identifying the specific mechanisms that govern their execution. Eye-tracking technology has emerged as a pivotal tool in this domain, which offers precise methods to analyze how visual information is processed and reflected in human behavior. By analyzing visual stimuli, eye-tracking studies provide insights into the complex interactions between visual perception, attention, and cognition.

Eye-tracking is particularly effective in quantifying visual attention, as it enables researchers to monitor gaze behaviors, such as fixations (pauses of the gaze on a specific point) and saccades (rapid eye movements between fixations). James [43] postulated that attention is inherently selective, with gaze directed toward stimuli of interest amid competing visual inputs. Furthermore, Yarbus [44] supported this foundational theory in a seminal study, which demonstrated that visual attention patterns vary according to the observer’s intent or context. More recently, Nadra et al. [45], Yan et al. [46], and Roth et al. [47] provided more precise quantifications of how gaze duration and intensity vary in the presence of high-interest stimuli. These findings have proven instrumental in understanding cognitive pathways, as they offer insights into how mental resources are selectively allocated to specific elements within a visual field.

The evolution of eye-tracking technologies has enabled their application in a diverse array of contexts, ranging from architecture to emotional cognition and urban design. For instance, Tang [48], who investigated the psychological impact of architectural spaces using fixed-location eye trackers, identified the ways in which spatial configurations influence human behavior. Skaramagkas et al. [49] extended this approach by incorporating neurophysiological measures to evaluate the interaction between visual attention and emotional stimuli, illustrating the utility of combining eye-tracking with techniques such as electroencephalography. Similarly, Gholami et al. [50], who analyzed urban park designs using wearable eye-tracking devices, revealed critical factors of visual perception and suggested practical improvements for urban planning.

Recent advancements in immersive technologies, such as virtual reality and AR, have further expanded the scope of human–computer interaction and behavioral analysis. These tools enable the simulation of realistic environments, allowing researchers to explore cognitive characteristics such as spatial reasoning, immersion, and decision-making in controlled yet dynamic contexts. In particular, AR has been increasingly utilized in applications requiring real-time pose estimation and movement tracking, contributing to advancements in behavioral biometrics and industrial human–computer interaction. Studies have introduced novel approaches for flexible human pose estimation [51], Gaussian coordinate encoding for efficient skeletal tracking [52], and asymmetric relation-aware learning for head pose estimatio. These methods enhance motion tracking and user interaction analysis [53], further supporting AR-based investigations into spatial reasoning and cognitive processes. Furthermore, AR has shown promise in investigating users’ perceptions and interactions with mixed-reality spaces by bridging the gap between real and virtual environments. Studies incorporating AR have provided insights into the cognitive processes underlying behaviors such as navigation, object manipulation, and spatial reasoning, thereby contributing to the development of practical applications in education and design [19,24].

In this study, we build upon these advancements by exploring how AR virtual spaces are cognitively processed compared with real-world environments. By employing eye-tracking technology, we investigate the visual and sensory mechanisms through which spatial characteristics in AR are perceived and translated into user behavior. Specifically, we observe gaze patterns, fixation metrics, and reaction times to evaluate how users collect and apply spatial information in AR environments. Furthermore, we assess the implications of AR technology for enhancing spatial thinking, decision-making, and design cognition, and potential applications in educational and professional contexts.

## 3. Research Methods and Process

### 3.1. Experiment Design and Participants

Our aim was to explore cognitive differences in spatial thinking during design processes facilitated by AR technology. Specifically, we focused on examining the effects of prior experience in spatial visualization training on user behavior and cognitive processes in AR-based spatial design tasks. Participants were classified into two distinct groups based on their educational background and prior exposure to spatial visualization learning, ensuring a clear differentiation in expertise levels. The experiment was conducted following ethical guidelines and was approved by the Institutional Review Board (IRB) (approval number: SSU-202107 HR-344-1). To ensure participant privacy and data security, all collected data were anonymized and stored securely, with access restricted to authorized researchers. Additionally, participants were informed of the study’s purpose, potential risks, and their right to withdraw at any time without penalty. Since AR-based tasks have the potential to induce cognitive overload or spatial disorientation, precautions were taken to mitigate these risks. The experimental sessions were structured to allow for adequate adaptation time before task execution, and participants were encouraged to take breaks if necessary. Research assistants were available to provide guidance and ensure that participants could engage with the AR environment comfortably. These measures helped maintain participant well-being while ensuring the validity of the collected data.

The experimental sessions for Group A (experimental group) were conducted over 17 days from 20 May to 5 June 2021, while Group B (control group) sessions took place over 18 days from 26 July to 12 August 2021. To maintain procedural consistency and minimize external variables, participants were kept in a separate waiting area from the experimental space before their sessions. This arrangement ensured that instructions and safety guidelines were communicated without any external interference. The experimental room was designed to provide a controlled environment, with dimensions measuring 1900 mm in width, 5000 mm in length, and 2700 mm in height.

Group A consisted of 56 participants who had formal training in 3D spatial visualization in design-related disciplines, such as architecture, interior architecture, and design. The average age of this group was 22.7 years (±1.8). Group B included 62 participants without any formal education or training in spatial design, whose average age was 25.1 years (±3.5). To confirm the validity of these classifications, participants were asked to complete a detailed online questionnaire during recruitment that documented their academic background and prior experience in spatial visualization tasks. This process ensured that the experimental group had a relevant educational foundation and that the control group did not.

**Figure 1:**
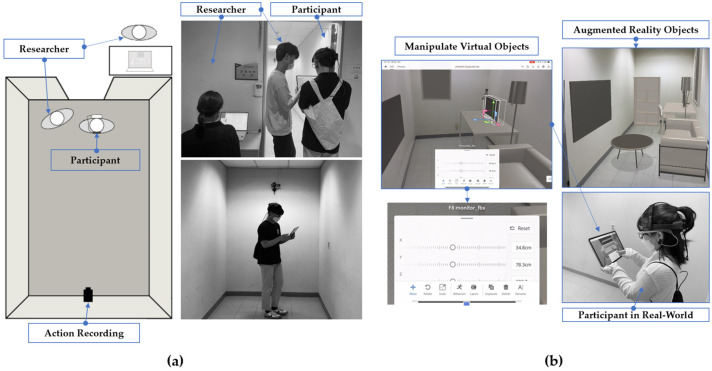
(**a**) Schematic representation of the experimental setup, including the positioning of eye-tracking equipment and augmented reality (AR) interfaces. The controlled environment ensured precise data collection for both groups. (**b**) Depiction of a participant performing the furniture arrangement task using a tablet to manipulate virtual objects rendered at actual size within a physical space. The immersive AR environment was designed to simulate real-world spatial tasks.

To establish an experimental framework, this study was conducted in multiple stages. Initially, a comprehensive literature review was performed to investigate the definitions and theoretical underpinnings of spatial thinking and spatial visualization. This review also included an analysis of prior studies and existing methodologies for measuring cognitive processes, which informed the development of the experimental design. This preliminary stage highlighted the role of educational experience in shaping spatial cognition and identified metrics for observation, such as gaze behaviors and interaction patterns, as critical indicators of cognitive performance. The experimental setup featured a 1:1 scale virtual environment created using AR technology ensuring realism and immersion in spatial tasks. Unlike traditional scaled-down modeling or two-dimensional visualization methods, the 1:1 scale AR environment allowed participants to perceive and interact with virtual objects at their actual size, maintaining accurate spatial relationships within the physical space. Virtual objects, such as furniture, were rendered at actual size within a physical space, which allowed participants to engage in authentic spatial decision-making tasks through AR interfaces delivered via tablets. A key feature of this AR system was real-time object manipulation, which enabled users to dynamically adjust, move, and rotate virtual objects within the environment. This real-time interaction allowed participants to test different spatial configurations instantly, improving their ability to evaluate depth, proportion, and arrangement within the space. Unlike traditional spatial visualization exercises, which often rely on static representations, this approach provided an interactive and iterative design experience. The primary task involved arranging virtual furniture in a predefined physical area while maintaining spatial coherence (see Figure 1). This setup ensured a controlled yet dynamic environment in which participants could engage directly with spatial design challenges, offering a more immersive alternative to conventional design exercises.

Before the main experiment, a pilot study was conducted with one participant from each group to validate the experimental design and procedures. This initial step enabled necessary adjustments to be performed to refine the methodology for ensuring the reliability and accuracy of data collection. During the main experiment, participants interacted with the AR environment while equipped with eye-tracking devices to capture real-time visual data. These devices recorded gaze metrics, primarily focusing on fixation duration, which provided insights into participants’ visual attention and cognitive processing. However, saccades and gaze patterns were not included in the analysis scope. Simultaneously, behavioral data were collected through observations of participants’ interactions with virtual objects, including their furniture arrangement strategies and decision-making processes.

Experiments for each group were conducted separately to minimize any potential cross-contamination of results. Throughout the sessions, participants performed spatial tasks requiring reasoning and decision-making, while we systematically collected data from eye-tracking and behavioral observations. Gaze data offered a detailed view of the allocation of visual attention during spatial tasks, while behavioral observations provided context for participants’ cognitive and physical interactions in the AR environment. Since this study primarily focuses on tracking learner adaptability within the AR environment based on prior experience, rather than system performance analysis, we did not measure specific AR parameters such as latency, rendering quality, or tracking accuracy. However, to ensure a consistent user experience and mitigate potential usability issues, participants received assistance from research assistants when needed. This support aimed to reduce cognitive load and allow participants to focus on their spatial tasks rather than on technical difficulties. While the study does not explore system-level optimizations, future research could examine the impact of these technical factors on cognitive processing and user interaction in AR environments.

### 3.2. Data Collection and Analysis Workflow

To investigate differences in spatial visualization ability based on participants’ design education backgrounds, a data collection methodology integrating eye-tracking technology and behavioral observation was employed. During the experiment, participants wore eye-tracking glasses to capture real-time gaze movements while engaging in AR-based spatial design tasks. The experiment was conducted in a hybrid physical–virtual environment in which AR objects, such as furniture, were rendered at a 1:1 scale and seamlessly integrated with the real-world space to enhance realism and immersion. This approach enabled the analysis of visual perception and behavioral interactions within an AR-assisted design workflow.

Prior to the experiment, each participant received instructions on how to manipulate AR content using a tablet (iPad Pro 12.9, Apple, Cupertino, CA, USA) and was given an opportunity to practice basic AR operations through a brief trial session. The main task required participants to design a learning space using 10 pieces of virtual furniture. They were allowed to move, rotate, and scale the objects freely to create their spatial arrangement, and when a participant finished their task, the experiment concluded. Given the differences in individual abilities related to space and interaction, the task was designed to allow participants to work comfortably. Throughout the experiment, visual attention and behavioral responses were continuously recorded. Eye-tracking data were collected using mobile eye-tracking equipment (Eye-Tracking Glasses 2 Wireless, SMI, Teltow, Germany), while behavioral interactions were captured using a high-resolution camcorder (SONY HDR-CX405, Sony Corporation, Tokyo, Japan).

**Figure 2:**
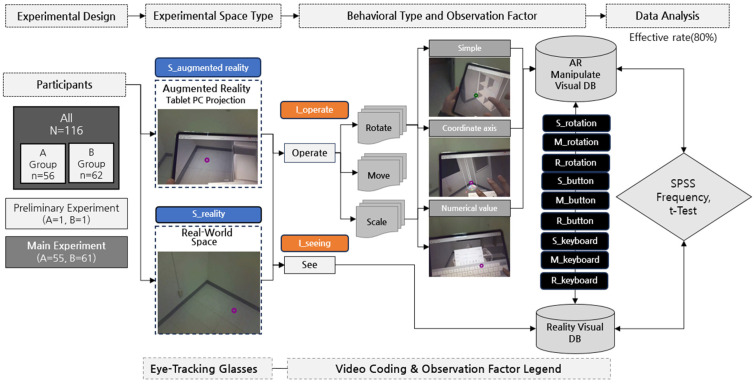
Experimental framework for analyzing visual perception and spatial interaction in AR-based design tasks.

Behavioral observations were systematically analyzed based on predefined behavioral criteria, following methodologies established in the literature [54]. The recorded data were coded and categorized into distinct behavioral observation types, which were then further subdivided into subcategories for detailed analysis. As illustrated in Figure 2, the experiment was designed to extract and analyze key behavioral factors influencing spatial visualization in AR. The behavioral data were classified into four primary categories: (1) Experiment Duration, which measured the total time taken by participants to complete the spatial design task; (2) Space Utilization, which examined how participants engaged with both real and AR environments during the visualization process. In this category, both quantitative and qualitative data were analyzed to assess participants’ spatial engagement. Specifically, we examined spatial movement patterns, interaction frequency with virtual elements, and time spent in different areas of the environment. These data were collected through motion tracking, behavioral observations, and user feedback to provide a comprehensive understanding of how participants navigated and interacted within the AR space; (3) Tablet Projection Interaction, which focused on two subcategories—Visual Attention (Seeing), referring to gaze behaviors directed at the AR interface, and Object Manipulation (Operating), capturing the spatial interaction processes; and (4) AR Virtual Space Interaction, which encompassed three primary behavioral actions: Move, Scale, and Rotate. These actions were further classified into three categories based on manipulation type: Coordinate Axis Manipulation (precise control along the X, Y, and Z axes), Numerical Input (direct value-based adjustments), and Simple Manipulation (intuitive, freehand interactions). This classification allowed for a more detailed analysis of how different interaction techniques were used within the AR environment.

To ensure the accuracy and reliability of data interpretation, behavioral observations were coded based on the frequency and duration of each identified behavior, with Move, Scale, and Rotate being measured in terms of their occurrences and the time spent on their execution. Frequency was defined as the number of occurrences of a particular behavioral action in the experimental session, whereas duration was measured as the time elapsed from the initiation of one behavioral action to the transition to another. For example, if a participant adjusted an object’s position multiple times within a session, each instance was counted as a separate occurrence in the frequency data. Similarly, the total time spent on moving, scaling, or rotating objects was recorded as duration. This systematic categorization facilitated an in-depth comparison of spatial interaction strategies between participants with and without prior design education.

For statistical analysis, SPSS Statistics 25 software was utilized to quantify and compare behavioral differences between the two participant groups. Independent samples *t*-tests were conducted to assess the statistical significance of variations in visual attention and spatial interaction behaviors. Frequency analysis enabled a comparative evaluation of behavioral patterns between groups, and mean values were interpreted based on absolute performance differences in spatial cognition. To facilitate relative comparisons, behavioral data were converted into percentage values, which allowed for a clearer understanding of how participants with different educational backgrounds approached spatial tasks in AR environments. By integrating visual perception analysis with behavioral observation, we developed a structured framework for evaluating cognitive differences in AR-assisted design processes. The findings contribute to an improved understanding of how users interact with AR environments based on their prior spatial training, and highlight key cognitive and behavioral attributes relevant to spatial reasoning, perception, and decision-making.

## 4. Results

### 4.1. Comparison of Experimental Time and Frequency Between Participant Groups

AR application in spatial design is crucial for developing effective user-centered design strategies, particularly for enhancing the understanding of human–computer interaction in virtual environments. Through this study, we aim to contribute to improving design education strategies that optimize user interaction in AR environments by employing behavior observation methodologies based on prior research [55,56]. The focus was on observing and analyzing user behaviors in AR-based virtual spaces.

As shown in Table 1, we compared the experimental participation time and frequency of participants with relevant expertise (Group A) and those without it (Group B). During the interactive experimental process, the frequency and duration of experiments were compared between the two groups. According to the frequency data, Group B (*M* = 190.7 s, *SD* = 78.9) exhibited a higher mean frequency than Group A (*M* = 165.0 s, *SD* = 69.2). This indicates that participants without prior design learning experience interacted more frequently and for longer durations during task execution.

In terms of experimental duration, the mean duration of Group B was longer (*M* = 621.6 s, *SD* = 224.5) than that of Group A (*M* = 552.3 s, *SD* = 176.5). This discrepancy may be attributed to differences in familiarity or proficiency with the tasks between the groups. Furthermore, Group B’s task duration range was more varied, with a wider spread (Min = 197.8, Max = 1177.4 s), than that of Group A (Min = 251 s, Max = 923.9 s). These results suggest greater variability in task completion time among participants in the control group (Group B), which may reflect differences in cognitive processing, decision-making speed, or task complexity experienced by them.

In summary, the comparative analysis highlights key differences in behavioral patterns based on prior design learning experience in AR-based virtual spaces. Participants without design experience exhibited a higher frequency of interactions and longer task durations, indicating a steeper learning curve when adapting to AR environments. These findings reinforce the importance of structured AR-based spatial training to bridge the gap between novice and experienced users, for ultimately enhancing spatial cognition and interaction efficiency.

### 4.2. Classification and Comparison of Key Behavioral Factors in AR Environments

#### 4.2.1. Visual Attention and Object Manipulation Interaction

**Figure 3:**
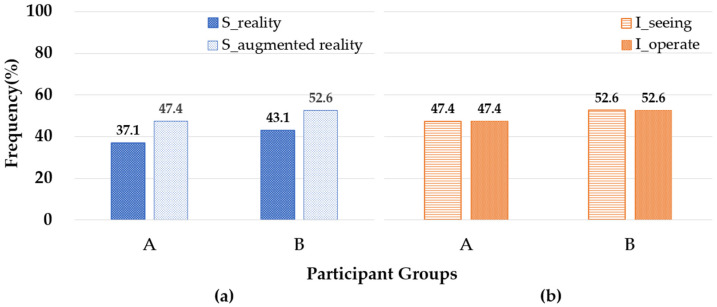
Comparison of behavioral observations between Groups A (*n* = 55) and B (*n* = 61) in AR and real-world environments: (**a**) Participants’ engagement in reality (S_reality) vs. AR (S_augmented reality) environments. (**b**) Participants’ visual attention (I_seeing) vs. interaction (I_operate).

A comparative analysis using behavioral observation data was conducted between the two participant groups in real and AR environments. The analysis focused on two dimensions: visual attention, which refers to how participants directed their gaze while interacting with AR content, and object manipulation, which measures their engagement with spatial transformations, such as moving, rotating, and scaling objects. The proportion of participant behaviors in real-world environments (S_reality) and AR environments (S_augmented reality) is presented in Figure 3.

For Group A, 37.1% of the participants demonstrated behavior in the real environment, while 47.4% interacted with the AR environment. In Group B, participation in the real environment accounted for 43.1%, whereas AR engagement was higher at 52.6%. This indicates a greater inclination toward AR interaction in Group B than in Group A (see Figure 3a). However, despite this difference in preference, both groups displayed similar behavioral patterns in terms of visual attention and object manipulation. Figure 3b provides a comparative analysis of participants’ visual attention (I_see)—which measures the time spent observing objects—and object manipulation interactions (I_operate) in real and AR environments. The results indicate that both Group A and Group B exhibited comparable interaction patterns, with 47.4% of participants focusing on visual attention and 52.6% engaging in object manipulation. These similarities suggest a consistent cognitive approach to spatial processing and interaction across both groups, regardless of prior design training.

In summary, this experiment examined the impact of design education background on participants’ ability to interact with AR environments. As shown in Figure 3a, the higher frequency of AR interactions among Group B participants—who lacked formal design experience—suggests that they may have relied more on exploratory interaction rather than structured spatial planning. Meanwhile, as depicted in Figure 3b, the similarities in visual attention and object manipulation patterns between the two groups indicate that fundamental cognitive processes related to observation and interaction remain relatively stable, regardless of educational background. This suggests that AR environments provide an intuitive spatial interaction experience that can be accessed effectively by users with varying levels of design expertise.

#### 4.2.2. Preferences for Object Manipulation Types

To compare the preferences for object manipulation types among participant groups in an AR environment, a frequency analysis was conducted. The analysis categorized behaviors into coordinate axis manipulation, numerical value manipulation, and simple manipulation, which included subcategories for resizing, moving, and rotating objects.

For coordinate axis manipulation, Group B exhibited a higher proportion of this behavior, with 51.7% performing resizing tasks and 48.3% engaging in rotation tasks. Group A showed slightly lower proportions, with 39.7% for resizing and 37.9% for rotation. These results suggest that participants in Group B, who were less familiar with spatial manipulation tasks in AR environments, may have relied more heavily than Group A participants on the visual tools provided by coordinate axes.

For numerical value manipulation, notable differences were observed between the two groups. Group A displayed a higher preference for precise numerical adjustments, with 45.7% engaging in resizing tasks and 9.5% in moving tasks. Group B participants showed a significantly lower preference for numerical input, with only 7.8% and 6.0% performing resizing and moving tasks, respectively; moreover, only 8.6% engaged in rotation tasks, further indicating their lower reliance on numerical adjustments.

These findings suggest that Group A participants, likely due to their experience in digital visualization, are more accustomed to using numerical values for precise object placement, while Group B participants, lacking formal design training, exhibited less engagement with fine-tuned adjustments.

**Figure 4:**
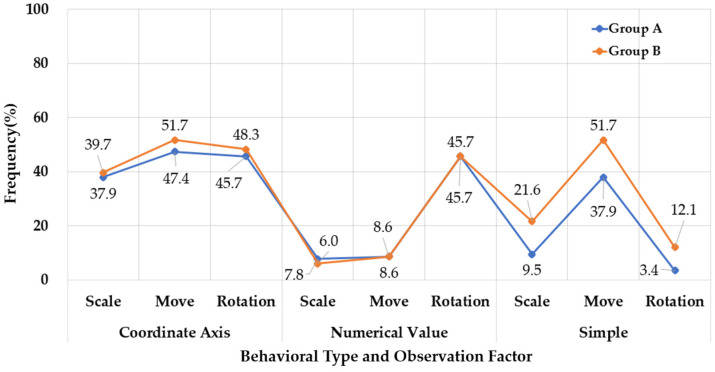
Comparison of object manipulation preferences between Groups A (*n* = 55) and B (*n* = 61) across different task dimensions in AR environments.

As regards the simple dimension, which evaluates basic object manipulations (resize, move, rotate), the two groups demonstrated differing preferences. Group B preferred moving objects (51.7%) to resizing (21.6%) and rotating (12.1%) them. However, Group A exhibited a slightly lower preference for rotation (3.4%) compared to 37.9% for movement and 45.7% for scaling. These differences suggest that participants without formal design training favor more intuitive and direct manipulation techniques, such as moving objects, whereas their trained counterparts show a stronger preference for controlled numerical input, reflecting advanced spatial reasoning and cognition (see Figure 4).

### 4.3. Statistical Significance of Design Learning Experiences in AR Environments

#### 4.3.1. Verification of Manipulation Frequency

To evaluate the differences in observed behavior data between the two groups, a *t*-test was conducted to assess significance. The experiment analyzed whether there were statistically significant differences in the frequency of behavioral observations for AR content manipulation methods—scaling (Scale), moving (Move), and rotating (Rotate)—with respect to behavioral observation elements, namely coordinate axis manipulation, numerical input manipulation, and simple manipulation (see Table 2).

Significant differences were observed in simple manipulation behaviors for the three behavioral observation types: Scale (*t* = −2.749, *p* < 0.01), Move (*t* = −4.243, *p* < 0.001), and Rotate (*t* = −2.451, *p* < 0.01). For numerical input manipulation, significant results were found only for Rotate (*t* = 2.422, *p* < 0.05). To further analyze the data, the absolute mean differences between the groups for each behavioral observation type were calculated, which identified the behaviors exhibiting the largest mean difference. Among simple manipulation behaviors, Scale and Move, which demonstrated significant differences, showed an absolute mean difference exceeding 1.7. The largest mean difference between the two groups (Group A: *M* = 7.5, *SD* = 7.3; Group B: *M* = 14.7, *SD* = 10.6) was for the Move behavior. Similarly, for the Scale behavior under simple manipulation, a significant mean difference was observed (Group A: *M* = 0.7, *SD* = 2.1; Group B: *M* = 3.2, *SD* = 6.7).

These results indicate that the educational background in design influences object manipulation in AR environments. The statistically significant differences observed in the dimensions of numerical input and simple manipulation suggest that participants with design-related educational backgrounds tend to perform tasks requiring precision and simplicity more frequently. This finding aligns with the hypothesis that training in related fields enhances participants’ ability to perform complex spatial manipulations. In contrast, the absence of significant differences in coordinate axis manipulation implies that intuitive tasks in AR environments are universally accessible regardless of educational background.

#### 4.3.2. Verification of Duration by Behavior Type

An independent samples *t*-test was conducted to examine the significance of the duration of sub-behavior observation elements categorized by type. The analysis compared four major categories: interaction in real-world and AR environments, visual attention observation (See), and object manipulation (Operate) (see Table 3).

The analysis of mean differences in the duration of behaviors between the real-world and AR environments, based on spatial visualization learning experiences, revealed that Group A (*M* = 14.6, *SD* = 34.6) had higher values than Group B (*M* = 7.5, *SD* = 12.0) in the real-world environment. Conversely, in the AR environment, Group B (*M* = 616.4 s, 10 min 16.4 s, *SD* = 221.5) demonstrated higher values than Group A (*M* = 525.1 s, 8 min 45.1 s, *SD* = 175.2).

For the sub-behavior observation element of visual attention (See) during tablet projection, the duration difference between Group A (*M* = 92.8, *SD* = 74.1) and Group B (*M* = 92.2, *SD* = 77.2) was marginal at 0.6 s. However, in object manipulation (Operate), Group B (*M* = 520.3, *SD* = 181.7) exhibited a longer duration than Group A (*M* = 447.5, *SD* = 129.3), with a difference of 72.8 s. Significant differences were observed in the duration of the sub-behavior observation elements of AR (*t* = −2.445, *p* < 0.05) and Operate (*t* = −2.462, *p* < 0.05).

#### 4.3.3. Comparison of Duration for Object Manipulation

For the two participant groups, significance was examined across three key dimensions—coordinate axis manipulation, numerical input manipulation, and simple manipulation—in an AR environment through an independent samples *t*-test. Behavior observations were analyzed to determine differences between the two groups in the time spent on specific tasks within these dimensions (see Table 4).

In the behavioral observation type Rotate, the axis manipulation behavior element showed a significant difference between groups (*t* = −3.076, *p* < 0.01). Group A (*M* = 36.9, *SD* = 27.7) exhibited lower values than Group B (*M* = 58.7, *SD* = 45.5). For simple manipulation behavior, Group B (*M* = 6.3, *SD* = 19.9) had higher values than Group A (*M* = 0.6, *SD* = 2.6), and there was a significant between-group difference (*t* = −3.139, *p* < 0.05). In object movement during simple manipulation behavior, there was a significant difference between the groups (*t* = −3.844, *p* < 0.001), with Group B (*M* = 133.4, *SD* = 77.1) showing higher values than Group A (*M* = 77.6, *SD* = 79.1). Regarding axis manipulation behavior, Group A (*M* = 251.5, *SD* = 128.9) scored higher than Group B (*M* = 199.4, *SD* = 114.6), and there was a significant difference between the groups (*t* = −3.076, *p* < 0.05). Size adjustment during simple manipulation behavior also showed a significant difference (*t* = −32.189, *p* < 0.05), with Group B (*M* = 17.1, *SD* = 46.4) scoring higher than Group A (*M* = 3.2, *SD* = 9.0).

The mean difference in the absolute values of manipulation methods between participants was compared with the overall mean difference for the three types of behavior observations (19.2). The axis manipulation behaviors in the Move and Rotate observation types (Move Difference: 52.2; Rotate Difference: −21.8) and the simple manipulation behaviors in the Move type (Difference: −55.8) displayed larger values. In Move, the durations for axis manipulation (simple manipulation) behavior were longer for Group A (Group B). Even for the same AR content manipulation method (Move), differences in manipulation behaviors based on spatial reasoning were observed.

A difference in object placement duration was evident between the two groups in tasks involving axis manipulation and simplicity. Group B participants consistently required more time to complete these tasks, suggesting that they found object manipulation in an AR environment more challenging or were unfamiliar with it. Conversely, there was no significant difference between the groups for tasks involving numerical input, indicating that both groups were equally proficient in completing abstract tasks. These results highlight that familiarity and experience with spatial tasks can significantly influence performance. This finding suggests the importance of considering user backgrounds when designing educational or practical applications in AR environments.

## 5. Discussion and Conclusions

### 5.1. Potential of Augmented Reality as an Educational Tool and Significance of the Study

In this study, we explored how differences in spatial thinking, shaped by prior experience in spatial visualization education, influence object manipulation behavior in AR environments. This study was guided by the question: “How does users’ experience with spatial visualization education influence their spatial thinking and object manipulation in AR environments?” To address this question, we used an experimental framework incorporating eye-tracking and behavioral observation to analyze cognitive and interactional differences between design majors (Group A) and non-majors (Group B).

The integration of AR into educational environments holds significant promise, particularly in design education, a field in which spatial reasoning and visualization are key competencies. This study demonstrates that AR can enhance spatial thinking and problem-solving abilities by offering immersive learning experiences that bridge the gap between theoretical knowledge and practical application [27].

The results revealed that Group A participants demonstrated superior efficiency in manipulating objects within AR environments and thus completed spatial tasks more swiftly and effectively than Group B participants. These findings are consistent with those of Tiwari et al. [7], who highlighted that immersive technologies, such as AR, can significantly improve spatial skills and comprehension by providing dynamic, real-time interactions with 3D objects. Statistical analysis through an independent samples *t*-test indicated significant differences between the two groups in terms of behavioral observation patterns, particularly in object placement, rotation, and scaling behaviors. The findings suggest that participants with prior design education exhibited enhanced spatial reasoning and problem-solving abilities, likely due to their familiarity with spatial cognition and virtual object manipulation.

Interestingly, while both groups exhibited similar visual attention patterns—as evidenced by eye-tracking data—differences emerged in task adaptation times. Group B required significantly more time to adjust to the AR environment and exhibited greater variability in interaction strategies. This finding suggests that those without formal design training may face initial cognitive barriers when engaging with virtual spatial environments, which reinforces the importance of structured AR-based spatial training.

In line with these findings, AR emerges as a powerful tool to facilitate the development of spatial reasoning, particularly in fields requiring advanced spatial skills, such as architecture and design [15]. These results support theoretical perspectives on spatial thinking and learning [13,27], affirming that AR can serve as an effective educational tool for enhancing spatial cognition and design proficiency. This study highlights the pedagogical potential of AR in bridging the gap between theoretical spatial concepts and hands-on experiential learning. By integrating AR into design curricula, educators can provide more immersive and interactive learning experiences, which can foster a deeper comprehension of spatial relationships. This finding aligns with the call for the use of adaptive learning tools in AR, which have been shown to enhance user engagement and learning outcomes by personalizing the educational experience [9]. Furthermore, this study supports the growing literature on the effectiveness of AR in bridging the gap between abstract learning and practical application in complex disciplines.

### 5.2. Limitations and Future Research Directions

While this study provides valuable insights into AR-based spatial reasoning, several limitations should be acknowledged. One key limitation is the composition of the participant pool, which consisted exclusively of university students from either a design or non-design background. As a result, the generalizability of the findings may be restricted, as they do not account for professionals or individuals with varying levels of spatial expertise. Future research should expand participation to a more diverse demographic, including individuals with different educational and professional backgrounds, to assess the broader applicability of the findings. Comparative analyses between novice and expert users could also provide deeper insights into how prior experience influences spatial reasoning, interaction efficiency, and cognitive engagement in AR environments.

Another limitation of this study lies in its reliance on behavioral and visual attention data as primary indicators of cognitive engagement. While these metrics were systematically recorded, they may not fully capture the depth of cognitive processing and decision-making strategies involved in real-world AR interactions. Furthermore, this study primarily utilized quantitative behavioral data, which may have resulted in an underrepresentation of qualitative aspects such as user intent, cognitive load, and subjective spatial perception. Given the importance of these factors in understanding spatial reasoning in AR environments, future research should adopt a mixed-methods approach, integrating behavioral analysis with qualitative feedback and cognitive workload assessments. Additionally, this study focused primarily on short-term engagement metrics and immediate cognitive interactions within the AR environment. The long-term effects of AR on student learning outcomes, retention, and skill development remain unexplored. This study did not explicitly measure the long-term usability and adaptability of AR-based learning tools in educational settings. Investigating how students retain and apply AR-enhanced skills beyond the initial learning phase would provide deeper insights into the system’s effectiveness in real-world applications. A longitudinal study could further assess how sustained engagement with AR impacts cognitive load, motivation, and learning efficiency across different levels of expertise. By addressing these gaps, future research can further establish AR as an innovative educational tool that enhances both immediate learning experiences and long-term knowledge retention.

Furthermore, while this study utilized 1:1 scale AR rendering and real-time object manipulation to enhance spatial immersion, their specific impact on cognitive load, usability, and spatial reasoning was not explicitly measured. Although these features offered a highly interactive spatial experience, they may have also introduced challenges, such as increased cognitive demand due to continuous real-time adjustments. A more comprehensive investigation into how AR interaction techniques influence cognitive processing, spatial comprehension, and decision-making efficiency would further strengthen the understanding of AR’s role in education. A further consideration is the absence of a structured feedback loop for iterative system refinement. While this study effectively examined user interaction patterns, no systematic adjustments were made to the AR system based on participant feedback. Future research should integrate iterative design methodologies that incorporate user feedback at different stages of system development. This approach would enhance system usability, engagement, and overall effectiveness, ultimately leading to a more adaptive and user-centered AR learning environment.

To advance the field of AR-based cognitive research, future studies should explore more complex and dynamic AR learning environments. Incorporating real-time spatial transformations, collaborative design tasks, and adaptive interaction models could provide deeper insights into how AR supports spatial cognition across different user groups. Expanding AR scenarios beyond static object manipulation to include real-time problem-solving tasks could further enhance ecological validity and practical applicability. Furthermore, advancements in machine learning and AI-based analytics offer promising avenues for improving real-time cognitive load assessment and behavioral tracking. The integration of adaptive AR interfaces that respond to user proficiency levels could optimize learning experiences by providing personalized guidance and interaction mechanisms, making AR-based education more effective and accessible.

In conclusion, this study reinforces the notion that AR holds significant potential as a transformative tool for spatial reasoning education. By addressing current limitations—such as participant diversity, the scope of cognitive load assessment, the integration of user feedback for system refinement, and AR usability metrics—future research can unlock the full potential of AR in educational as well as professional applications. A more interdisciplinary approach to AR-based learning design will ensure greater accessibility, adaptability, and effectiveness in diverse learning environments.

## Figures and Tables

**Table 1:** Comparison of frequency and duration of interactions during experiment performance between participant groups (*N* = 116).

Type	Frequency		Duration	
	A	B	A	B
*N* (%)	55 (47.4)	61 (52.6)	55 (47.4)	61 (52.6)
*M* (%)	165.0 (46.4)	190.7 (53.6)	552.3 (47.0)	621.6 (53.0)
*SD* (%)	69.2 (46.8)	78.9 (53.2)	176.5 (55.9)	224.5 (44.1)
Min	64.0	69.0	251.1	197.8
Max	417.0	482.0	923.9	1177.4

A: Experimental group, B: Control group. *N* = Participant Number (%), *M*, *SD*, Min, Max = Seconds (%).

**Table 2:** Statistical comparison of object manipulation frequencies between groups across different task dimensions in augmented reality (AR) environments.

Manipulation Type			*M*	*SD*	*t*	*p*	Diff.
Coordinate axis	scale	A	4.7	4.1	−1.286	0.201	−1.2
		B	5.9	5.8			
	move	A	14.1	6.4	−0.201	0.841	−0.3
		B	14.4	7.6			
	rotate	A	6.3	3.0	−1.660	0.100	−1.3
		B	7.5	4.8			
Numerical value	scale	A	0.3	1.1	0.937	0.351	0.1
		B	0.2	0.6			
	move	A	0.3	0.6	−0.829	0.409	−0.2
		B	0.4	1.4			
	rotate	A	6.1	2.5	2.422 *	0.017	1.3
		B	4.7	3.4			
Simple	scale	A	0.7	2.1	−2.749 **	0.007	−2.6
		B	3.2	6.7			
	move	A	7.5	7.3	−4.243 ***	0.000	−7.2
		B	14.7	10.6			
	rotate	A	0.1	0.5	−2.451 *	0.016	−0.7
		B	0.8	2.0			

* *p* < 0.05, ** *p* < 0.01, *** *p* < 0.001. Unit: Frequency. A = Experimental group (*n* = 55), B = Control group (*n* = 61). The values for Diff. (Difference) represent the difference value for the control group based on the value for the experimental group.

**Table 3:** Comparison of duration between groups in experimental environments and object manipulation interactions.

Type		*M*	*SD*	*t*	*p*	Diff.
Real World	A	14.6	34.6	1.498	0.137	7.1
	B	7.5	12.0			
Augmented reality	A	525.1	175.2	−2.445 *	0.016	−91.3
	B	616.4	221.5			
See	A	92.8	74.1	0.042	0.967	0.6
	B	92.2	77.2			
Operate	A	447.5	129.3	−2.462 *	0.015	−72.8
	B	520.3	181.7			

* *p* < 0.05, Unit: sec. A = Experimental group (*n* = 55), B = Control group (*n* = 61). The values for Diff. (Difference) represent the difference value for the control group based on the value for the experimental group.

**Table 4:** Comparison of object manipulation performance in AR environments based on spatial visualization experience.

Manipulation Type			*M*	*SD*	*t*	*p*	Diff.
Coordinate axis	scale	A	35.4	32.7	−1.221	0.225	−10.3
		B	45.7	54.4			
	move	A	251.5	128.9	2.308 *	0.023	52.2
		B	199.4	114.6			
	rotate	A	36.9	27.7	−3.076 **	0.003	−21.8
		B	58.7	45.5			
Numerical value	scale	A	3.4	12.9	1.172	0.244	2.1
		B	1.3	4.6			
	move	A	1.7	4.2	−1.273	0.206	−2.3
		B	4.0	12.8			
	rotate	A	44.5	23.1	1.607	0.111	9.0
		B	35.5	35.0			
Simple	scale	A	3.2	9.0	−2.189 *	0.031	−13.9
		B	17.1	46.4			
	move	A	77.6	79.1	−3.844 ***	0.000	−55.8
		B	133.4	77.1			
	rotate	A	0.6	2.6	−2.139 *	0.035	−5.8
		B	6.3	19.9			

* *p* < 0.05, ** *p* < 0.01, *** *p* < 0.001. Unit: sec. A = Experimental group (*n* = 55), B = Control group (*n* = 61). The values for Diff. (Difference) represent the difference value for the control group based on the value for the experimental group. 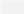: The difference value is greater than the absolute average of 19.2.

## Data Availability

Data sharing is not applicable to this article.
